# Comparative Analysis of CO_2_ Adsorption Performance of Bamboo and Orange Peel Biochars

**DOI:** 10.3390/molecules30071607

**Published:** 2025-04-03

**Authors:** Choul Woong Kwon, Sungho Tae, Soumen Mandal

**Affiliations:** 1 Department of Smart City Engineering, Graduate School, Hanyang University ERICA, 1271 Sa-3-dong, Sangnok-gu, Ansan 15588, Republic of Korea; hottrinity@hanyang.ac.kr; 2 School of Architecture and Architectural Engineering, Hanyang University ERICA, 1271 Sa-3-dong, Sangnok-gu, Ansan 15588, Republic of Korea; 3 Industry-University Cooperation Foundation, Hanyang University ERICA, 1271 Sa-3-dong, Sangnok-gu, Ansan 15588, Republic of Korea; sou.chm@gmail.com

**Keywords:** carbon capture and storage (CCS), biochar, BET surface area, CO_2_ adsorption efficiency, thermogravimetric analysis

## Abstract

Carbon capture and sequestration (CCS) is an essential strategy for mitigating greenhouse gas emissions and addressing climate change. In this study, the biochar of bamboo and orange peel (BB and OPB) are synthesized and appraised as potential CO_2_ adsorbents. Comprehensive characterizations viz. sorption isotherm, FTIR spectroscopy, and SEM-EDS reveal substantial differences in their structural and functional properties. OPB exhibits a significantly higher BET surface area (40.13 m^2^/g) compared to BBs (7.38 m^2^/g). FTIR and EDS analyses further demonstrate more amine, carboxylic, ester, and ether functional groups in OPB, indicating its affinity for CO_2_ molecules. The CO_2_ adsorption isotherm shows a higher adsorption capacity (22.83 cm^3^/g) in OPB than BB (14.12 cm^3^/g) at 273 K and 1 bar. The adsorption process is augmented by mesoporous structures and interactions between surface functional groups and CO_2_ molecules. The thermogravimetric analysis further reveals the higher CO_2_ uptake capability of OPB than BB. This result also shows that the CO_2_ uptake stabilizes after 48 h for both the biochars. These results highlight the potential of OPB as an efficient CCS material, demonstrating the importance of specific biochar properties in the development of CO_2_ capture.

## 1. Introduction

With increasing impacts of climate change observed around the globe, reducing the emissions of carbon dioxide (CO_2_) to reach carbon neutrality has received a great deal of attention [1,2,3]. Carbon neutrality or net-zero carbon dioxide emissions is when the amount of CO_2_ emitted and the amount removed from the atmosphere is equilibrated, to mitigate global warming. Innovative technologies and natural solutions for CCS are essential in this context [1]. Highly promising for CO_2_ capture and permanent carbon decomposition and storage, an emerging solution is biochar, a carbon-rich material derived from the pyrolysis of biomass [4,5].

Biochar is a versatile and eco-friendly environmental solution. Biochar has recently gained attention for its multifaceted properties and environmental benefits [3,6,7]. Biochar was originally known for its ability to improve soil quality [8,9,10,11], but it has also recently been identified as a potential carbon management tool because of its porous structure and large surface area which enable it to readily adsorb and retain CO_2_ [3,12,13,14]. Biochar produced from renewable biomass resources like agriculture residues, forestry waste, and organic matter offers a sustainable and low-cost opportunity to reduce atmospheric CO_2_ levels [13,15,16,17]. In addition, being stable and not easily decomposed, it is excellent medium for long-term carbon sequestration, which assists in achieving carbon neutrality objectives [18,19,20]. The task ahead to achieve carbon neutrality is colossal. CO_2_ emissions from industrial processes, transportation, and energy generation have reached alarming levels, and the need for technological and nature-based carbon capture solutions has never been greater [21,22]. High-cost and energy-intensive, conventional CCS methods, including direct air capture (DAC) [23,24,25] and geological carbon capture and storage (CCS) [26,27], are widely researched, and implemented but have a very high energy and cost requirements [28,29]. Currently, there are almost no solutions which can at scale sequester carbon and more, while biochar is an established, nature-based, low-energy solution which provides not only climate, but a variety of other natural benefits, including increased soil fertility [30] and water retention [31], as well as a perfect waste management [4] solution.

The way in which biochar sequesters CO_2_ depends on its particular physicochemical features [3,32,33]. The porous nature of biochar offers multiple surfaces for CO_2_ to bind to, while its chemical properties, which depend on the biomass and the pyrolysis conditions, help it bind well with CO_2_ molecules [34]. Biochar thus becomes both an effective sink for CO_2_ and a solid carrier of it because of its dual ability to physically adsorb and chemically bond CO_2_. Biochar can also continue to adsorb atmospheric CO_2_ over time when used in soils, extending its implications for carbon sequestration [35].

While promising, biochar as a tool for carbon capture is still in its early stages and optimizing its functionality will require significant research. The effectiveness of CO_2_ adsorption by biochar is influenced by the feedstock material used, the temperature of pyrolysis, and surface modification procedures [3]. In addition, scaling biochar even for broad application in carbon sequestration presents challenges with regards to feedstock availability, production cost, and environmental footprint. However, due to its potential for long-term carbon sequestration, biochar is gaining attention as a potential aspect of carbon neutrality strategies, especially when paired with other carbon capture technology or land management approaches.

Focusing on the potential of using biochar as an adsorbent of CO_2_, this study aims to evaluate the potential of the biochar of bamboo (BB) and orange peel (OPB) as sustainable and efficient materials for CCS applications. The selection of bamboo and orange peel as raw materials for biochar is based on their abundance, sustainability, and unique physicochemical properties. Bamboo grows rapidly, has a high lignocellulosic content, and is widely used in biochar studies, while orange peel is an agro-industrial waste rich in amine [36], oxygen-containing functional groups [37], making it a promising adsorbent. The pyrolysis temperature of the biomasses is selected at 500 °C based on the literature as biochar production at this temperature is reported to show the balance between yield, pH, surface area, pore development, and functional group retention [38,39,40,41]. Several studies have demonstrated that 500 °C acts as a critical pyrolysis temperature for enhancing the sorption capacity of biochar due to significant changes in its functional groups and structural properties. Specifically, modifications in amide groups, phosphate ions (PO_4_^3−^), and aromatic ring structures contribute to the improved adsorption efficiency of biochar [42,43]. Furthermore, increasing the pyrolysis temperature leads to an elevation in biochar pH and carbon content, while volatile elements such as hydrogen, oxygen, nitrogen, and sulfur are progressively eliminated as the pyrolysis process advances [44]. Chatterjee et al. further reported that biochars produced at 600–700 °C exhibit higher carbon and ash content, a reduction in nitrogen content, and a significant enhancement in surface area and pore volume, which are essential factors in improving biochar’s adsorption properties [45]. Therefore, efforts are made to understand how surface area, porosity, and functional group composition influence CO_2_ adsorption capacity. Biochars are synthesized and characterized using Brunauer–Emmett–Teller (BET) N_2_ sorption isotherms to determine surface area and pore size distribution. SEM has been used to examine the surface morphologies of the biochars. FTIR and EDS are employed to examine chemical composition and functional group distributions. Weight loss profiles by TGA is used to evaluate CO_2_ releasing from biochars after exposure to CO_2_ chamber. This study intends to contribute to the growing body of knowledge on the role of biochar in mitigating climate change and advancing strategies for a more sustainable, carbon-neutral future.

## 2. Results and Discussion

### 2.1. Surface Area and Pore Structure

**Figure 1:**
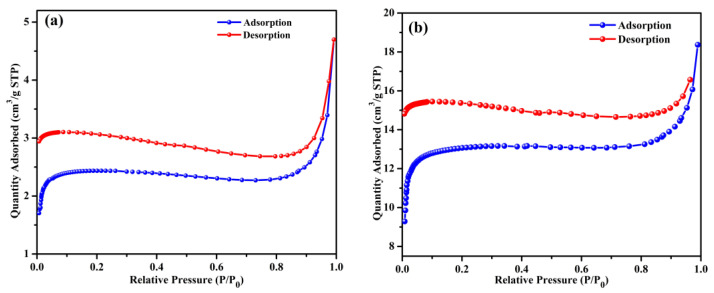

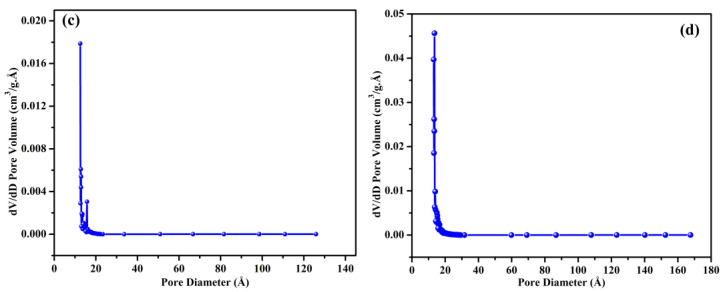
N_2_ adsorption–desorption isotherms (at 77 K) of BB (**a**) [21], OPB [46] (**b**), and pore size distribution (BJH method) of BB (**c**) [21] and OPB (**d**) [46].

The morphological properties, including the specific surface area and porosity, of BB and OPB are assessed using N_2_ adsorption/desorption analysis at 77 K. The N_2_ adsorption–desorption plots (Figure 1a,b) and BJH pore size distribution plots (Figure 1c,d) are regenerated from our earlier published work [21,46].

The BB and OPB samples demonstrate adsorption capacities of 4.70 cm^3^/g and 18.37 cm^3^/g, respectively, at a relative pressure (P/P_0_) of 0.989. This adsorption pattern suggests the presence of mesoporous structures. The observed adsorption behavior for both samples align with a Type I(b) isotherm curve. At low relative pressures (below 0.02), a sharp increase in adsorption is observed, which can be attributed to the micropore filling. As the relative pressure increases, the behavior transitions to multilayer adsorption combined with capillary condensation, indicating the coexistence of macropores and mesopores [21,46,47].

Single-point surface areas at P/P_0_ = 0.29 are measured as 7.3794 m^2^/g for BB and 40.1305 m^2^/g for OPB. Using the BET method, the surface areas are determined to be 9.7213 m^2^/g and 51.63 m^2^/g, respectively. The average pore diameters calculated using the BJH adsorption method are 14.095 Å for BB and 14.94 Å for OPB.

### 2.2. FTIR Spectroscopy

**Figure 2:**
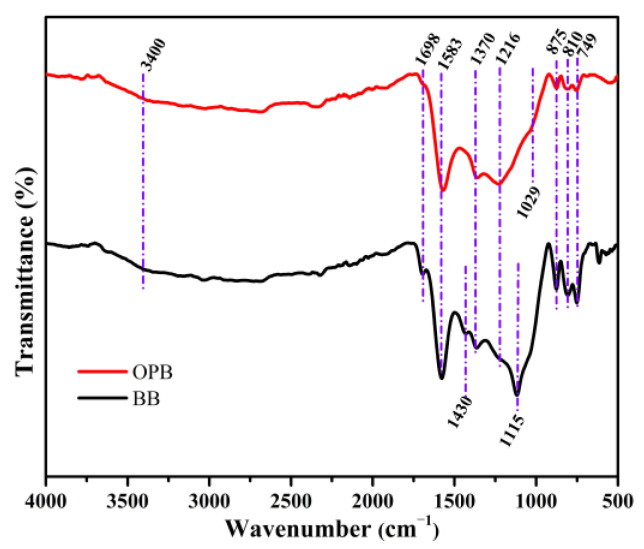
FTIR spectra of OPB and BB.

The FTIR spectra of BB and OPB are presented in Figure 2. It can be seen that except for three absorption peaks in FTIR, almost all the peaks are similar in both the synthesized biochars. The band at 3400 cm^−1^ indicates the O–H stretching vibration. The stretching vibration by the carbonyl groups of the carboxyl groups can be related to the peak at 1698 cm^−1^ [48,49]. Vibrational peaks at 1583 and 1115 cm^−1^ are ascribed to the C=O stretching vibration and C=O symmetrical stretching, respectively [50]. Aromatic C=C vibrations can also be attributed to the absorption peak at 1583 cm^−1^ [51]. The bending of C–H in aliphatic hydrocarbon has resulted in the FTIR peak at 1430 cm^−1^ [52]. The C–O stretching of carboxylic, ester, and ether groups in cellulose and hemicellulose is realized from the absorption peak at 1029 cm^−1^ [48,52]. The acidic functional groups from carboxylic and phenolic groups that have generated the peak at 1370 cm^−1^ as well as the presence of amine groups can be evidenced from the appearance of the absorption peak at 1216 cm^−1^ [53]. It is clearly evidenced that the presence of functional amine groups in OP biochar is much higher in comparison to BB. The absorption spectra attributed to the carbonates are found at 875 cm^−1^ [52]. The peak at 810 and 749 cm^−1^ in the FTIR spectrum of biochar is associated with C–H bending vibrations. This indicates the presence of aliphatic hydrocarbons in the biochar structure [50]. From the comparison of FTIR results, it is evident that OPB contains more carboxylic, ester, and ether groups. In addition, BB shows the presence of more aliphatic hydrocarbon at 1430 cm^−1^. Therefore, it can be concluded from the FTIR results that OPB contains more amine, carboxylic, ester, and ether groups.

### 2.3. Scanning Electron Microscopy and Energy-Dispersive X-Ray Spectroscopy

**Figure 3:**
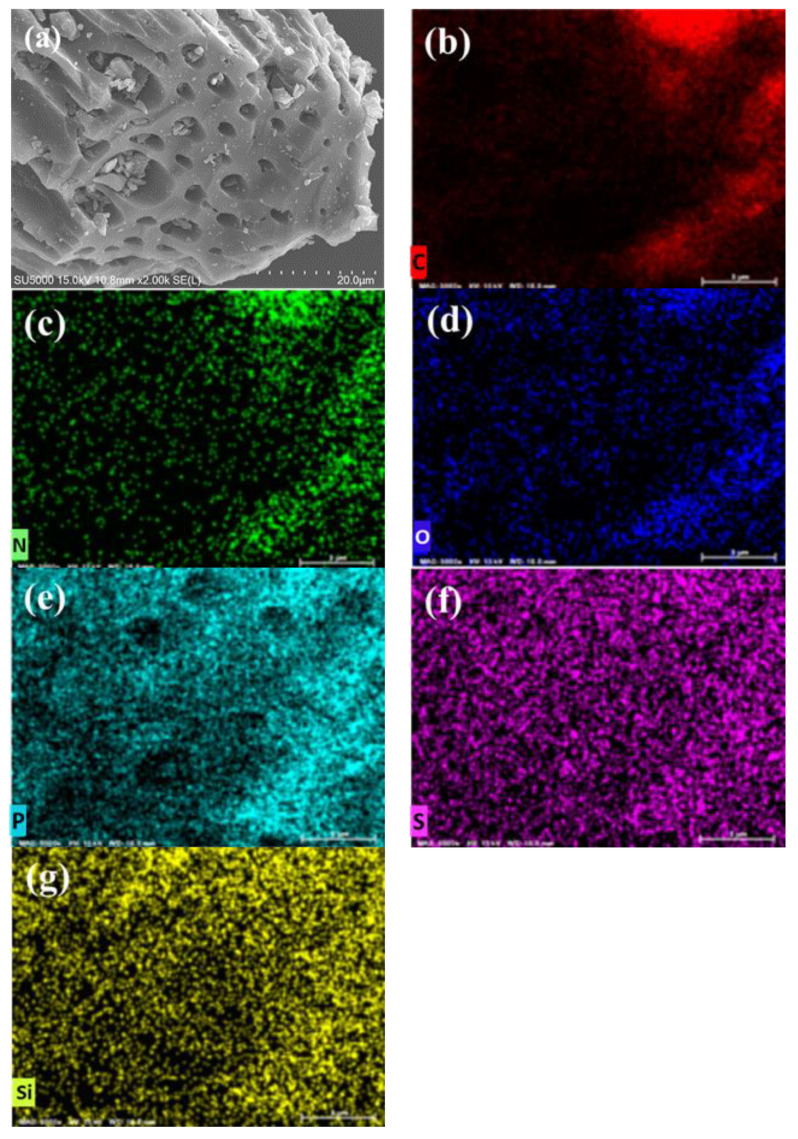
(**a**) SEM and (**b**–**g**) elemental distribution of C, N, O, P, S, and Si in BB sample by EDS mapping.

**Figure 4:**
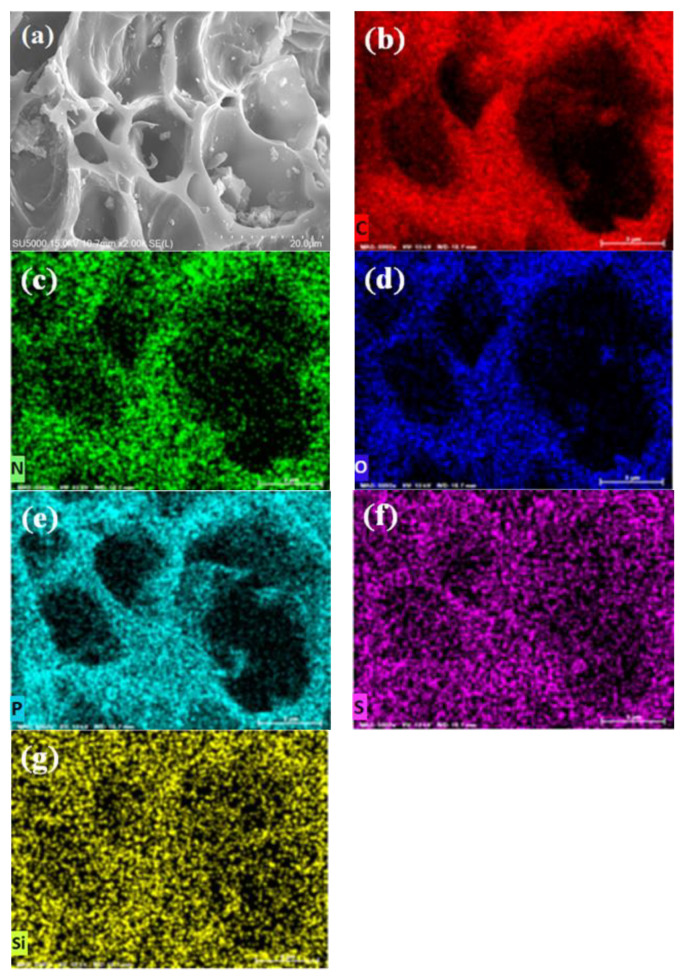
(**a**) SEM and (**b**–**g**) elemental distribution of C, N, O, P, S, and Si in OPB sample by EDS mapping.

The morphologies of BB and OPB samples are examined using FE-SEM and the micrographs are presented in Figure 3a and Figure 4a, respectively. The elemental mapping is also carried out to estimate the presence of the probable surface functional groups on the biochar surfaces. From the SEM micrographs of BB and OPB, it can be seen that the biochars have porous and channel-like morphologies. However, on comparison with the same scale, the pores in the OPB (Figure 4a) appear to have much bigger diameters than the BB (Figure 3a) sample.

On the EDS mapping, both the samples show the presence of C, N, S, Si, P, and O elements on the surfaces. The obtained quantities of the elements by EDS mapping are presented in Table 1. It can be clearly observed from the EDS mapping of BB and OPB that the content of carbon (Figure 3b and Figure 4b) is almost the same for both samples. However, the presence of P, S, and Si is much higher in the BB sample (Figure 3e–g) in comparison to OPB (Figure 4e–g). In addition, OPB (Figure 4c,d) contains N and O in much higher amounts than BB (Figure 3c,d).

The EDS results clearly validate the findings in the FTIR results that the presence of carboxylic, ester, and amine groups are much higher in the OPB samples than BB.

### 2.4. CO_2_ Adsorption Characteristics

**Figure 5:**
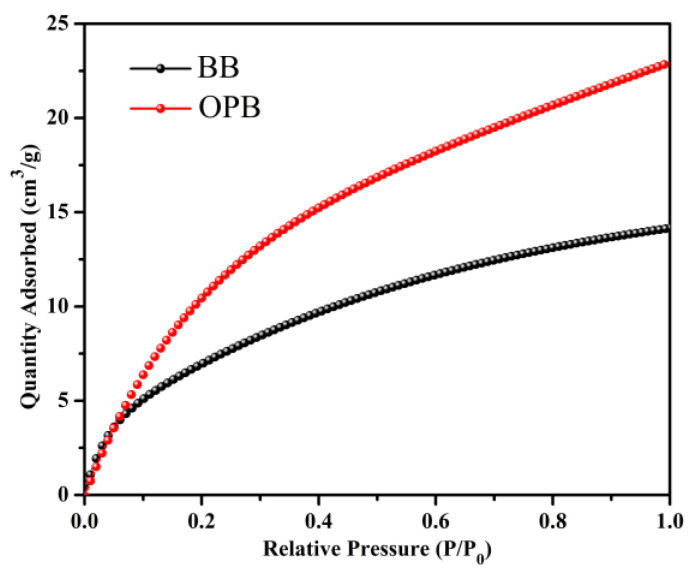
Carbon dioxide adsorption isotherm at 273 K.

The CO_2_ adsorption properties of the porous BB and OPB biochars are thoroughly investigated by measuring their CO_2_ sorption isotherms at 273 K, with the resulting adsorption isotherm data shown in Figure 5. The observed up-convex nature of the isotherm curves demonstrates the strong interaction between the porous samples and CO_2_ molecules, which can be attributed to their mesoporous structural characteristics. This structural feature enhances the accessibility of adsorption sites and facilitates the diffusion of CO_2_ molecules within the material. In both cases, the CO_2_ uptake is increased consistently with rising pressure, indicating a favorable adsorption process. Notably, the adsorption has not reached a saturation point within the experimental pressure range of up to 1 bar.

Quantitatively, the CO_2_ adsorption capacity of the OPB sample was determined to be 22.83 cm^3^/g, substantially higher than the 14.12 cm^3^/g recorded for the BB sample. This disparity in adsorption capacity can be ascribed to the following two critical factors: (i) the surface area differences: The BET surface area analysis, by N_2_ sorption isotherms, has revealed a considerable difference in surface area between the two samples. The OPB sample has exhibited a surface area of 40.1305 m^2^/g, significantly surpassing the 7.3794 m^2^/g measured for the BB sample. A larger surface area correlates directly with the availability of active adsorption sites, thereby enhancing the adsorption performance of the OPB material. For gas adsorption applications, the formulation of biochars with highly porous structures and a large specific surface area is crucial [54,55,56,57]; (ii) the functional group contributions: The chemical composition and surface functional groups of the biochars are characterized using FTIR and EDS, revealing significant differences in their surface chemistry. The OPB exhibits a noticeable presence of amine (-NH_2_) functional groups, which strongly interact with CO_2_ through chemisorption [32,34,58]. These interactions primarily occur via the formation of carbamate species, where CO_2_ reacts with surface amines to form stable bonds, enhancing the adsorption capacity. This mechanism plays a crucial role in applications where chemisorption dominates over physisorption. Additionally, FTIR analysis identifies a higher concentration of carboxyl (-COOH) and ester (-COOR) functional groups in OPB. These oxygen-containing functional groups facilitate further CO_2_ adsorption through physisorption mechanisms, including hydrogen bonding and dipole–dipole interactions. The presence of these functional groups not only enhances adsorption through multiple interaction pathways but also improves the overall adsorption efficiency and selectivity of the OPB. The synergistic effect of these functional groups, combining strong chemical interactions with additional physical adsorption sites, explains the superior CO_2_ uptake observed for OPB.

In contrast, the BB sample’s lower adsorption capacity can be attributed to its smaller surface area and the relatively lower abundance of functional groups that favor CO_2_ adsorption. These findings are consistent with prior studies emphasizing the role of chemical modifications, particularly amine functionalization, in enhancing CO_2_ adsorption performance [59,60,61,62]. Such functionalization techniques are widely regarded as effective methods for improving the adsorption characteristics of porous materials. Furthermore, the integration of amine groups and other functional groups through either inherent composition or post-synthetic modifications provides a robust strategy for optimizing materials for CO_2_ capture and storage applications.

**Figure 6:**
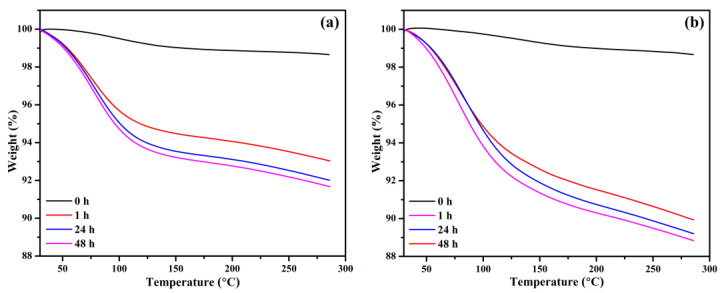
Thermogravimetric data of (**a**) BB and (**b**) OPB, after exposure at CO_2_ chamber for different durations.

Further investigation of the BB and OPB samples is conducted using thermogravimetric analysis (TGA) after exposure to a CO_2_ chamber. Prior to CO_2_ exposure, the samples are placed in a vacuum chamber for 6 h to ensure the removal of all pre-adsorbed gases and moisture. The exposure durations in the CO_2_ chamber are systematically varied at 0, 1, 24, and 48 h intervals. Beyond 48 h of exposure, the TGA weight loss (%) stabilizes, indicating that additional exposure time does not significantly impact the adsorption characteristics. The TGA plots for BB (Figure 6a) and OPB (Figure 6b) reveal that the biochars without CO_2_ exposure exhibit negligible weight losses of 1.35% and 1.34%, respectively. These minimal weight changes are attributed to minor gas and moisture adsorption during sample handling and loading into the TGA instrument. In contrast, significant weight losses are observed after CO_2_ exposure. For BB, the weight losses after 1, 24, and 48 h of exposure are 6.97%, 7.99%, and 8.32%, respectively. Similarly, for OPB, the corresponding weight losses are 10.07%, 10.80%, and 11.16%.

These findings suggest that the CO_2_ adsorption process is most active during the initial hours of exposure and approaches a saturation point by 48 h. The higher weight losses observed for OPB compared to BB can be attributed to the larger surface area and the abundance of functional groups in OPB, which enhance its CO_2_ adsorption capacity. The data highlight the distinct adsorption and capacity differences between the two biochars under identical experimental conditions. The superior performance of the OPB highlights the importance of optimizing surface area and chemical functionalization in designing biochars for effective CO_2_ capture.

To compare the CO_2_ adsorption efficiencies of the BB and OPB samples, a few other materials from the earlier literature are tabulated in Table 2 with their processing and modification methods and CO_2_ adsorption efficiencies. The table highlights the variations in the CO_2_ adsorption capacity of biochars derived from different biomass sources under diverse pyrolysis conditions, activation methods, and post-treatment processes. Among the reported biochars, perilla biochar pyrolyzed at 700 °C showed the highest CO_2_ uptake of 2.312 mmol/g without any activation or post-treatment, indicating its inherent porosity and adsorption potential [63]. Similarly, biochars derived from Miscanthus, switchgrass, corn stover, and sugarcane bagasse exhibited significantly improved CO_2_ adsorption capacities when subjected to physical and chemical activation followed by pyrolysis at 700 °C, reaching a maximum of 2.89 mmol/g, highlighting the influence of activation techniques on adsorption efficiency [45]. On the other hand, biochars from coffee grounds, pine sawdust, and oak demonstrated lower adsorption capacities, ranging from 0.14 to 0.73 mmol/g, suggesting that biomass type and pyrolysis conditions play a critical role in CO_2_ capture efficiency. The role of activation agents was particularly evident in the case of bagasse and rice husk biochars activated with ZnCl_2_, which exhibited CO_2_ adsorption capacities of 1.74 mmol/g and 1.29 mmol/g, respectively [64], showing the effectiveness of chemical activation in enhancing the porosity and surface area.

This study highlights the KOH-activated OPB, which achieved a CO_2_ adsorption capacity of 1.01 mmol/g, demonstrating that alkaline activation enhances CO_2_ capture by increasing pore volume. The BB synthesized in this study showed an adsorption capacity of 0.63 mmol/g, which, while lower than some other biochars, still demonstrated competitive performance, especially considering that it was produced at moderate pyrolysis temperatures without post-modification This highlights the potential of OPB and BB as a cost-effective and environmentally sustainable option, as the production process requires less energy and avoids additional chemical treatments, reducing both economic and environmental burdens.

## 3. Materials and Methods

### 3.1. Biochar Preparation

One biochar was synthesized from the bamboo (*Bambusa vulgaris*) stem and another from orange peels from the oranges procured from the markets in Ansan, South Korea. Prior to further processing, biomasses were cut into small pieces. Both the collected biomass materials were cleaned with purified water followed by deionized (DI) water. Washed biomasses were then dried inside an electric oven at a set temperature of 100 °C for 48 h. Then, the biomasses were pulverized separately into powder using electric grinder (CUCKOO Electronics, Soul, South Korea). The ground powders were mechanically sieved to obtain the homogenous sized powder of 50 mesh size. For the activation of the biomasses, the collected powders were mixed separately with 10% aqueous potassium hydroxide (KOH) maintaining a mixing ratio of 1 kg to 1 L. The KOH-treated biomasses were kept for 10 h at ambient temperature and then pyrolyzed individually using an electrical muffle furnace (MSF-22, Lab House, Gyeonggi-do, Republic of Korea). The pyrolysis was carried out in oxygen-restricted conditions at 500 °C with a heating rate of 10 °C/min and isothermal condition of 1 h at 500 °C. After furnace cooling, pyrolyzed biomasses were taken out of the furnace and blended separately with 2% aqueous hydrochloric acid (HCl) for 2 h. Subsequently, the biochar samples were washed several times using warm DI water while waiting to attain a neutral pH.

### 3.2. Characterizations

The pore structures and specific surface areas of the biochars were analyzed using a Brunauer–Emmett–Teller (BET) N_2_ adsorption isotherm analyzer (Model: 3Flex 5.01, Micromeritics, Norcross, GA, USA). Measurements were performed at 77 K within a relative pressure range of 0 to 1.0, following a degassing process lasting over 12 h. The same instrument has been used to study the CO_2_ adsorption–desorption studies at 273 K.

The functional groups present in the synthesized biochar samples were characterized using FT-IR spectroscopy (Perkin Elmer UATR Two, Shelton, CT, USA). The morphology of the synthesized BB and OPB samples was analyzed using a field emission scanning electron microscope (FE-SEM, Hitachi S-4800, Hitachi, Tokyo, Japan) operating at an accelerating voltage of 15 kV. Additionally, an energy-dispersive X-ray spectroscopy (EDS) attachment integrated with the FE-SEM was employed to investigate the surface composition of the biochars. Thermogravimetry analyses were performed using a simultaneous thermal analyzer (PerkinElmer STA 6000, PerkinElmer, Waltham, MA, USA).

## 4. Conclusions

This study investigates the CO_2_ adsorption properties of bamboo biochar (BB) and orange peel biochar (OPB), emphasizing their morphological, chemical, and adsorption properties. BB and OPB are synthesized and examined for CO_2_ adsorption. The results showed significant differences in their adsorption capacities and surface characteristics. OPB exhibits a larger surface area (40.13 m^2^/g), higher pore volume, and a greater abundance of functional groups, including amine, carboxylic, ester, and ether groups, compared to BB. That contributes to the higher CO_2_ adsorption capacity of OPB (22.83 cm^3^/g) as compared with BB (14.12 cm^3^/g).

The results from the FTIR and EDS analyses highlighted the critical role of functional groups in enhancing chemisorption interactions with CO_2_, making OPB a possible superior adsorbent due to its rich chemical composition. The SEM micrographs further confirm OPB’s larger pore diameters, which improve gas diffusion and adsorption efficiency. TGA analysis indicates that CO_2_ adsorption ensues most actively within the initial hours and stabilizes after 48 h. However, at all the durations, OPB demonstrates better CO_2_ intakes in comparison to BB.

It can be realized from this study that optimizing surface area, porosity, and types of functional group present in biochar is crucial for the CO_2_ adsorption performance. The superior CO_2_ adsorbent performance of OPB indicates it as a promising material for CO_2_ capture and storage applications. This study establishes the significance of both the structural and chemical properties for the applications of biochars in advanced adsorption technologies, particularly in the context of carbon capture and storage (CCS) solutions.

## Figures and Tables

**Table 1:** Elemental analysis of biochars by EDS.

Biochar ID	Element	C	N	S	Si	P	O
BB	Mass (%)	68.70	11.85	0.83	0.92	11.08	6.62
OPB	Mass (%)	66.08	17.21	0.34	0.01	4.74	11.62

**Table 2:** CO_2_ adsorption capacities of biochars made from different feedstocks.

Sl. No.	Biomass	Pyrolysis Temperature (°C)	Activation Agent	Post-Treatment of Biochar	CO_2_ Intake (mmol/g)	Ref.
1.	Pine sawdust	550	-	-	0.67	[65]
2.	Pine sawdust	550	Steam	-	0.73	[65]
3.	Coffee grounds	400	-	-	0.14	[12]
4.	Coffee grounds	400	-	3-Aminopropy-ltrimethoxysilane	0.41	[12]
5.	Coffee grounds	400	-	HCl, K_2_Cr_2_O_7_	0.46	[12]
6.	Bagasse	500	ZnCl_2_	-	1.74	[64]
7.	Rice husk	500	ZnCl_2_	-	1.29	[64]
8.	Soybean	600	ZnCl_2_	CO_2_ Physical activation	0.93	[66]
9.	Coconut Shell	800		CO_2_ activation	1.09	[67]
10.	Perilla	700	-	-	2.312	[63]
11.	Korean oak	400	-	-	0.597	[63]
12.	Japanese oak	500	-	-	0.379	[63]
13.	Soybean stover	700	-	-	0.707	[63]
14.	Vine shoots	500	-	With CO_2_ at 800 °C	1.02	[68]
15.	Wheat straw	600	-	With CO_2_ at 800 °C	1.30	[68]
16.	Miscanthus, switchgrass, corn stover, sugarcane bagasse	500	-	-	0.4–0.49	[45]
		600	-		0.75–0.82	
		700	-		0.78–0.93	
		800	-		0.59–0.67	
17.	Miscanthus, switchgrass, corn stover, sugarcane bagasse	500	-	Physical activation in low-frequency ultrasound then chemical activation with tetraethylene-pentamime	0.86–1.23	[45]
		600	-		2.15–2.53	
		700	-		2.22–2.89	
		800	-		1.34–1.74	
18.	Orange peel	500	KOH	-	1.01	This study
19.	Bamboo	500	KOH	-	0.63	This study

## Data Availability

Data will be available on request.

## References

[1] Yang S., Yang D., Shi W., Deng C., Chen C., Feng S. (2023). Global evaluation of carbon neutrality and peak carbon dioxide emissions: Current challenges and future outlook. Environ. Sci. Pollut. Res..

[2] Schraven D., Joss S., De Jong M. (2021). Past, present, future: Engagement with sustainable urban development through 35 city labels in the scientific literature 1990–2019. J. Clean. Prod..

[3] Amer N.M., Lahijani P., Mohammadi M., Mohamed A.R. (2024). Modification of biomass-derived biochar: A practical approach towards development of sustainable CO_2_ adsorbent. Biomass Convers. Biorefinery.

[4] Mandal S., Ishak S., Adnin R.J., Lee D.-E., Park T. (2023). An approach to utilize date seeds biochar as waste material for thermal energy storage applications. J. Energy Storage.

[5] Mandal S., Ishak S., Lee D.-E., Park T. (2022). Optimization of eco-friendly *Pinus resinosa* biochar-dodecanoic acid phase change composite for the cleaner environment. J. Energy Storage.

[6] Al Masud M.A., Shin W.S., Sarker A., Septian A., Das K., Deepo D.M., Iqbal M.A., Islam A.R.M.T., Malafaia G. (2023). A critical review of sustainable application of biochar for green remediation: Research uncertainty and future directions. Sci. Total Environ..

[7] Amalina F., Krishnan S., Zularisam A., Nasrullah M. (2023). Recent advancement and applications of biochar technology as a multifunctional component towards sustainable environment. Environ. Dev..

[8] Hu Q., Jung J., Chen D., Leong K., Song S., Li F., Mohan B.C., Yao Z., Prabhakar A.K., Lin X.H. (2021). Biochar industry to circular economy. Sci. Total Environ..

[9] Diatta A.A., Fike J.H., Battaglia M.L., Galbraith J.M., Baig M.B. (2020). Effects of biochar on soil fertility and crop productivity in arid regions: A review. Arab. J. Geosci..

[10] Liang M., Lu L., He H., Li J., Zhu Z., Zhu Y. (2021). Applications of biochar and modified biochar in heavy metal contaminated soil: A descriptive review. Sustainability.

[11] Chen J., Lü S., Zhang Z., Zhao X., Li X., Ning P., Liu M. (2018). Environmentally friendly fertilizers: A review of materials used and their effects on the environment. Sci. Total Environ..

[12] Liu S.-H., Huang Y.-Y. (2018). Valorization of coffee grounds to biochar-derived adsorbents for CO_2_ adsorption. J. Clean. Prod..

[13] Zhang Y., Qu M., Li J., Ren L., Wang F., Wang J., Yang F., Cheng F. (2024). Relationship of CO_2_ adsorption performance and physicochemical property of biochar prepared by different types of biomass waste. J. Environ. Chem. Eng..

[14] Zhang T., Xiong Z., Zhao Y., Zhang J. (2025). Comparative study on the adsorption performance of CO_2_ and Hg in flue gas using corn straw and pine biochar modified by KOH. Sep. Purif. Technol..

[15] Younas M., Sohail M., Leong L., Bashir M.J., Sumathi S. (2016). Feasibility of CO_2_ adsorption by solid adsorbents: A review on low-temperature systems. Int. J. Environ. Sci. Technol..

[16] Francis J.C., Nighojkar A., Kandasubramanian B. (2023). Relevance of wood biochar on CO_2_ adsorption: A review. Hybrid Adv..

[17] Igalavithana A.D., Choi S.W., Dissanayake P.D., Shang J., Wang C.-H., Yang X., Kim S., Tsang D.C., Lee K.B., Ok Y.S. (2020). Gasification biochar from biowaste (food waste and wood waste) for effective CO_2_ adsorption. J. Hazard. Mater..

[18] Afshar M., Mofatteh S. (2024). Biochar for a sustainable future: Environmentally friendly production and diverse applications. Results Eng..

[19] Kuzyakov Y., Bogomolova I., Glaser B. (2014). Biochar stability in soil: Decomposition during eight years and transformation as assessed by compound-specific 14C analysis. Soil Biol. Biochem..

[20] Wang J., Xiong Z., Kuzyakov Y. (2016). Biochar stability in soil: Meta-analysis of decomposition and priming effects. Gcb Bioenergy.

[21] Mandal S., Ishak S., Ariffin M.A.M., Lee D.-E., Park T. (2023). Effect of pore structure on the thermal stability of shape-stabilized phase change materials. J. Mater. Res. Technol..

[22] Mandal S., Ishak S., Singh J.K., Lee D.-E., Park T. (2022). Synthesis and application of paraffin/silica phase change nanocapsules: Experimental and numerical approach. J. Energy Storage.

[23] Sodiq A., Abdullatif Y., Aissa B., Ostovar A., Nassar N., El-Naas M., Amhamed A. (2023). A review on progress made in direct air capture of CO_2_. Environ. Technol. Innov..

[24] Nemet G.F., Callaghan M.W., Creutzig F., Fuss S., Hartmann J., Hilaire J., Lamb W.F., Minx J.C., Rogers S., Smith P. (2018). Negative emissions—Part 3: Innovation and upscaling. Environ. Res. Lett..

[25] Bisotti F., Hoff K.A., Mathisen A., Hovland J. (2024). Direct Air capture (DAC) deployment: A review of the industrial deployment. Chem. Eng. Sci..

[26] Lin Z., Kuang Y., Li W., Zheng Y. (2024). Research status and prospects of CO_2_ geological sequestration technology from onshore to offshore: A review. Earth-Sci. Rev..

[27] Yu X., Catanescu C.O., Bird R.E., Satagopan S., Baum Z.J., Lotti Diaz L.M., Zhou Q.A. (2023). Trends in research and development for CO_2_ capture and sequestration. ACS Omega.

[28] McLaughlin H., Littlefield A.A., Menefee M., Kinzer A., Hull T., Sovacool B.K., Bazilian M.D., Kim J., Griffiths S. (2023). Carbon capture utilization and storage in review: Sociotechnical implications for a carbon reliant world. Renew. Sustain. Energy Rev..

[29] Wang Q., Du C., Zhang X. (2024). Direct air capture capacity configuration and cost allocation based on sharing mechanism. Appl. Energy.

[30] Nepal J., Ahmad W., Munsif F., Khan A., Zou Z. (2023). Advances and prospects of biochar in improving soil fertility, biochemical quality, and environmental applications. Front. Environ. Sci..

[31] Razzaghi F., Obour P.B., Arthur E. (2020). Does biochar improve soil water retention? A systematic review and meta-analysis. Geoderma.

[32] Guo S., Li Y., Wang Y., Wang L., Sun Y., Liu L. (2022). Recent advances in biochar-based adsorbents for CO_2_ capture. Carbon Capture Sci. Technol..

[33] Zhang C., Ji Y., Li C., Zhang Y., Sun S., Xu Y., Jiang L., Wu C. (2023). The application of biochar for CO_2_ capture: Influence of biochar preparation and CO_2_ capture reactors. Ind. Eng. Chem. Res..

[34] Qiao Y., Wu C. (2022). Nitrogen enriched biochar used as CO_2_ adsorbents: A brief review. Carbon Capture Sci. Technol..

[35] Gui X., Xu X., Zhang Z., Hu L., Huang W., Zhao L., Cao X. (2025). Biochar-amended soil can further sorb atmospheric CO_2_ for more carbon sequestration. Commun. Earth Environ..

[36] Koochi Z.H., Jahromi K.G., Kavoosi G., Ramezanian A. (2023). Fortification of Chlorella vulgaris with citrus peel amino acid for improvement biomass and protein quality. Biotechnol. Rep..

[37] Zhengfeng S., Ming C., Geming W., Quanrong D., Shenggao W., Yuan G. (2023). Synthesis, characterization and removal performance of Cr(VI) by orange peel-based activated porous biochar from water. Chem. Eng. Res. Des..

[38] Zhang L., Ren Y., Xue Y., Cui Z., Wei Q., Han C., He J. (2020). Preparation of biochar by mango peel and its adsorption characteristics of Cd (II) in solution. RSC Adv..

[39] Kundu S., Khandaker T., Anik M.A.-A.M., Hasan M.K., Dhar P.K., Dutta S.K., Latif M.A., Hossain M.S. (2024). A comprehensive review of enhanced CO_2_ capture using activated carbon derived from biomass feedstock. RSC Adv..

[40] Sangon S., Kotebantao K., Suyala T., Ngernyen Y., Hunt A.J., Supanchaiyamat N. (2024). ZnCl_2_ activated mesoporous carbon from rice straw: Optimization of its synthetic process and its application as a highly efficient adsorbent for amoxicillin. Environ. Sci. Water Res. Technol..

[41] Islam M.N., Sarker J., Khatton A., Hossain S.M., Sikder H.A., Ahmed R., Chowdhury A.S. (2022). Synthesis and characterization of activated carbon prepared from jute stick charcoal for industrial uses. Sch. Int. J. Chem. Mater. Sci..

[42] Xu X., Cao X., Zhao L. (2013). Comparison of rice husk-and dairy manure-derived biochars for simultaneously removing heavy metals from aqueous solutions: Role of mineral components in biochars. Chemosphere.

[43] Shen X., Zeng J., Zhang D., Wang F., Li Y., Yi W. (2020). Effect of pyrolysis temperature on characteristics, chemical speciation and environmental risk of Cr, Mn, Cu, and Zn in biochars derived from pig manure. Sci. Total Environ..

[44] Zhang J., Liu J., Liu R. (2015). Effects of pyrolysis temperature and heating time on biochar obtained from the pyrolysis of straw and lignosulfonate. Bioresour. Technol..

[45] Chatterjee R., Sajjadi B., Chen W.-Y., Mattern D.L., Hammer N., Raman V., Dorris A. (2020). Effect of pyrolysis temperature on physicochemical properties and acoustic-based amination of biochar for efficient CO_2_ adsorption. Front. Energy Res..

[46] Mandal S., Ishak S., Lee D.-E., Park T. (2022). Shape-stabilized orange peel/myristic acid phase change materials for efficient thermal energy storage application. Energy Rep..

[47] Qi L., Tang X., Wang Z., Peng X. (2017). Pore characterization of different types of coal from coal and gas outburst disaster sites using low temperature nitrogen adsorption approach. Int. J. Min. Sci. Technol..

[48] Siipola V., Tamminen T., Källi A., Lahti R., Romar H., Rasa K., Keskinen R., Hyväluoma J., Hannula M., Wikberg H. (2018). Effects of Biomass Type, Carbonization Process, and Activation Method on the Properties of Bio-Based Activated Carbons. https://bioresources.cnr.ncsu.edu/resources/effects-of-biomass-type-carbonization-process-and-activation-method-on-the-properties-of-bio-based-activated-carbons/.

[49] Sarmah A.K., Srinivasan P., Smernik R.J., Manley-Harris M., Antal M.J., Downie A., van Zwieten L. (2010). Retention capacity of biochar-amended New Zealand dairy farm soil for an estrogenic steroid hormone and its primary metabolite. Soil Res..

[50] Dong Y., Yu Y., Wang R., Chang E., Hong Z., Hua H., Liu H., Jiang J., Xu R. (2022). Insights on mechanisms of aluminum phytotoxicity mitigation by canola straw biochars from different regions. Biochar.

[51] Behazin E., Ogunsona E., Rodriguez-Uribe A., Mohanty A.K., Misra M., Anyia A.O. (2016). Mechanical, chemical, and physical properties of wood and perennial grass biochars for possible composite application. BioResources.

[52] Choi Y.-K., Srinivasan R., Kan E. (2020). Facile and economical functionalized hay biochar with dairy effluent for adsorption of tetracycline. ACS Omega.

[53] Thongsamer T., Vinitnantharat S., Pinisakul A., Werner D. (2022). Chitosan impregnation of coconut husk biochar pellets improves their nutrient removal from eutrophic surface water. Sustain. Environ. Res..

[54] Shafawi A.N., Mohamed A.R., Lahijani P., Mohammadi M. (2021). Recent advances in developing engineered biochar for CO_2_ capture: An insight into the biochar modification approaches. J. Environ. Chem. Eng..

[55] Khandaker T., Hossain M.S., Dhar P.K., Rahman M.S., Hossain M.A., Ahmed M.B. (2020). Efficacies of carbon-based adsorbents for carbon dioxide capture. Processes.

[56] Presser V., McDonough J., Yeon S.-H., Gogotsi Y. (2011). Effect of pore size on carbon dioxide sorption by carbide derived carbon. Energy Environ. Sci..

[57] Deng S., Wei H., Chen T., Wang B., Huang J., Yu G. (2014). Superior CO_2_ adsorption on pine nut shell-derived activated carbons and the effective micropores at different temperatures. Chem. Eng. J..

[58] Soo X.Y.D., Lee J.J.C., Wu W.-Y., Tao L., Wang C., Zhu Q., Bu J. (2024). Advancements in CO_2_ capture by absorption and adsorption: A comprehensive review. J. CO2 Util..

[59] Yaumi A., Bakar M.A., Hameed B. (2018). Melamine-nitrogenated mesoporous activated carbon derived from rice husk for carbon dioxide adsorption in fixed-bed. Energy.

[60] Dissanayake P.D., You S., Igalavithana A.D., Xia Y., Bhatnagar A., Gupta S., Kua H.W., Kim S., Kwon J.-H., Tsang D.C. (2020). Biochar-based adsorbents for carbon dioxide capture: A critical review. Renew. Sustain. Energy Rev..

[61] Shen W., Fan W. (2013). Nitrogen-containing porous carbons: Synthesis and application. J. Mater. Chem. A.

[62] Guo T., Ma N., Pan Y., Bedane A.H., Xiao H., Eić M., Du Y. (2018). Characteristics of CO_2_ adsorption on biochar derived from biomass pyrolysis in molten salt. Can. J. Chem. Eng..

[63] Sethupathi S., Zhang M., Rajapaksha A.U., Lee S.R., Mohamad Nor N., Mohamed A.R., Al-Wabel M., Lee S.S., Ok Y.S. (2017). Biochars as potential adsorbers of CH_4_, CO_2_ and H2S. Sustainability.

[64] Boonpoke A., Chiarakorn S., Laosiripojana N., Towprayoon S., Chidthaisong A. (2011). Synthesis of activated carbon and MCM-41 from bagasse and rice husk and their carbon dioxide adsorption capacity. J. Sustain. Energy Env..

[65] Igalavithana A.D., Choi S.W., Shang J., Hanif A., Dissanayake P.D., Tsang D.C., Kwon J.-H., Lee K.B., Ok Y.S. (2020). Carbon dioxide capture in biochar produced from pine sawdust and paper mill sludge: Effect of porous structure and surface chemistry. Sci. Total Environ..

[66] Thote J.A., Iyer K.S., Chatti R., Labhsetwar N.K., Biniwale R.B., Rayalu S.S. (2010). In situ nitrogen enriched carbon for carbon dioxide capture. Carbon.

[67] Son S.-J., Choi J.-S., Choo K.-Y., Song S.-D., Vijayalakshmi S., Kim T.-H. (2005). Development of carbon dioxide adsorbents using carbon materials prepared from coconut shell. Korean J. Chem. Eng..

[68] Manyà J.J., García-Morcate D., González B. (2020). Adsorption performance of physically activated biochars for postcombustion CO_2_ capture from dry and humid flue gas. Appl. Sci..

